# Antioxidant, Anti-Inflammatory and Anti-Angiogenic Properties of *Citrus lumia* Juice

**DOI:** 10.3389/fphar.2020.593506

**Published:** 2020-12-03

**Authors:** Antonella Smeriglio, Marcella Denaro, Valeria D’Angelo, Maria Paola Germanò, Domenico Trombetta

**Affiliations:** Department of Chemical, Biological, Pharmaceutical and Environmental Sciences, University of Messina, Messina, Italy

**Keywords:** citrus juice, antioxidant, anti-inflammatory, anti-angiogenic, polyphenols, ascorbic acid, chick chorioallantoic membrane, zebrafish embryos

## Abstract

*Citrus* juices are a rich source of bioactive compounds with various and well-known health benefits. The aim of this study was to investigate the polyphenols and ascorbic acid content as well as to investigate the antioxidant, anti-inflammatory and anti-angiogenic properties of the juice of an ancient Mediterranean species, *Citrus lumia* Risso (CLJ). The antioxidant and anti-inflammatory activities were evaluated by several *in vitro* cell-free and cell-based assays, whereas two different *in vivo* models, the chick chorioallantoic membrane (CAM) and the zebrafish embryos, were used to characterize the anti-angiogenic properties. Twenty-eight polyphenols were identified by RP-LC-DAD-ESI-MS analysis (flavonoids 68.82% and phenolic acids 31.18%) with 1-caffeoyl-5-feruloylquinic acid and kaempferol 3′-rhamnoside, which represent the most abundant compounds (25.70 and 23.12%, respectively). HPLC-DAD analysis showed a high ascorbic acid content (352 mg/kg of CLJ), which contributes with polyphenols to the marked and dose-dependent antioxidant and anti-inflammatory properties observed. CLJ showed strong and dose-dependent anti-angiogenic activity as highlighted by the inhibition of blood vessel formation on CAMs and the decrease of endogenous alkaline phosphatase on zebrafish embryos. Moreover, within the concentration range tested, no dead or malformed embryos were recorded. Certainly, further studies are needed to investigate the molecular mechanisms underlying these promising biological effects, but considering the evidence of the present study, the use of CLJ as a ready-to drink safe prevention strategy for inflammatory-based diseases correlated to angiogenesis could be justified.

## Introduction

Given the changes occurring in lifestyle and the growing evidences, which suggest a direct correlation between Mediterranean diet and improved health, international guidelines for health promotion and disease prevention recommend to consume mainly functional foods such as fruits and vegetables (Slavin and Lloyd, 2012). However, despite there is a global consensus about the protective role of fruits and vegetables, there is no clear evidence about the effects of consuming the fruit juices (Hyson, 2015), because their lower fiber content and higher caloric density in comparison with the fresh fruits. On the contrary, it has been demonstrated that fruit juices without additives such as the sweeteners, labelled by FDA as 100% *Citrus* juice, retain the majority of nutrients as well as of other non-nutrients bioactive compounds of the parent fruit such as ascorbic acid and polyphenols, well-known for their health effects (Hyson, 2015).

*Citrus* juices are widely distributed and commonly consumed beverages worldwide, because *Citrus* fruits are easy to find and “ready to drink”, providing a natural balance of water, vitamins, minerals, sugars, carotenoids, fiber and organic acids, together with a pleasant and fresh flavour (Hyson, 2015).

Moreover, *Citrus* juice have been identified as one of the most promising sources of flavonoids in the human diet, compounds well-known for their antioxidant, cytoprotective, anti-angiogenic, anti-inflammatory and anti-cancer properties (Barreca et al., 2017; Cirmi et al., 2017).

Several studies have established that diet, along with early screening, play a pivotal role in the management of several inflammation-based diseases, suggesting that prevention starts from what we eat and what we drink (Rossi et al., 2014). However, the translation of *in vitro* and *in vivo* results on clinic scenario still represents a difficult obstacle to overcome.

Despite the unfavourable weather has led to smaller crops in the last year in several countries, current worldwide production of *Citrus* fruits settles around 92 million tons, with the oranges which represent alone about the half of the total production, followed by tangerines/mandarins, grapefruits and lemons/limes (United States Department of Agriculture, 2020a). However, *Citrus* crops are not limited to mainstream species, because this genus is fairly variegated and constantly evolving, comprising several species, varieties, cultivars and hybrids (Barreca et al., 2020). Only a small part of this production is assigned to the fresh market, while the other part is mainly addressed to make fresh juices, *Citrus*-based drinks, marmalades, jelly and flavouring ingredients (Smeriglio et al., 2018a).

Recently, we have focused our attention on an ancient Mediterranean *Citrus* fruit, *Citrus lumia* Risso, (Smeriglio et al., 2018a; Smeriglio et al., 2019a). The chemical composition, and the antioxidant, anti-cholinesterase, and neuroactive properties of its essential oil were investigated, showing marked antioxidant and neuroactive effects, paving the way for its potential application in the field of oxidative-based neurodegenerative diseases (Smeriglio et al., 2018a). Moreover, the extract of *C. lumia* albedo revealed marked antioxidant and free radical scavenging properties as well as significant cytoprotective activity on tert-butyl hydroperoxide-treated lymphocytes, suggesting that this by-product is a valuable source for nutraceutical employment (Smeriglio et al., 2019a).

Considering this, the aim of the present study has been to investigate for the first time the chemical features, in particular the polyphenol profile and the ascorbic acid content, of fresh *C. lumia* juice, focusing on the evaluation of its antioxidant, anti-inflammatory and anti-angiogenic properties.

## Materials and Methods

### Chemicals

The Folin-Ciocalteu reagent, sodium carbonate (Na_2_CO_3_), aluminium chloride (AlCl_3_), sodium nitrite (NaNO_2_), sodium hydroxide (NaOH), *m*-phosphoric acid, 1,1-diphenyl-2-picrylhydrazyl (DPPH), 2,20-azino-bis(3-ethylbenzothiazoline-6-sulfonic acid (ABTS), potassium persulfate (K_2_S_2_O_8_), 6-hydroxy-2,5,7,8-tetramethylchromane-2-carboxylic acid (Trolox), 2,2′-azobis(2-methylpropionamidine) dihydrochloride (AAPH), fluorescein sodium salt, sodium phosphate dibasic (Na_2_HPO_4_), potassium phosphate monobasic (KH_2_PO_4_), 2,4,6-tris(2-pyridyl)-S-triazine (TPTZ), iron sulphate heptahydrate (FeSO_4_ • 7H_2_O), sodium acetate (CH_3_COONa), bovine serum albumin (BSA) heat shock fraction protease, fatty acid and essentially globulin free (pH 7, ≥98%), trypsine from porcine pancreas Type IX-S lyophilized powder (13,000–20,000 BAEE units/mg protein), perchloric acid, trizma-base, retinoic acid, diethanolamine, *p-*nitrophenyl phosphate disodium salt, ethylenediaminetetraacetic acid (EDTA), butylated hydroxytoluene (BHT), diclofenac sodium, lipopolysaccharides from *Escherichia coli* O55:B5, 2′,7′-dichlorofluorescein diacetate (DCF-DA), hydrogen peroxide, Histopaque®-1077, RPMI-1640 with L-glutamine, penicillin-streptomycin solution, fetal bovine serum and trypan blue were purchased from Sigma-Aldrich (MSt. Louis, MO, United States). Acetonitrile, trifluoroacetic acid and formic acid were HPLC grade and were purchased, as well as L-ascorbic acid solution 1.0 mg/ml in acetonitrile:water, certified reference material Cerilliant^®^ and DMSO, from Merck (Darmstadt, Germany).

### Plant Material and Sample Preparation

Fresh fruits of *C. lumia* Risso were harvested in March 2019 by a local farmer in Agrigento (Sicily, Italy). *C. lumia* Risso juice (CLJ) was obtained by a manually juice processor and centrifuged at 3500 × g (Neya 10R, REMI, Mumbai, India) for 15 min at 4°C. The supernatant, which density at 20°C is 1.011 g/cm^3^, was collected and stored at −80°C until use. CLJ was properly diluted in deionized water before *in vitro* and *in vivo* analyses, whereas for HPLC analysis CLJ was diluted in MilliQ water and filtered by a 0.22 µm nylon syringe filter.

### Total Phenols Content

Total phenols were determined according to Smeriglio et al. (2018b). Fifty microliters of sample solution (6.33–50.6 µl/6.25–50 mg/ml of CLJ diluted in deionized water) or gallic acid as reference compound (75–600 µg/ml), were added to Folin-Ciocalteu (500 µl) and deionized water (450 µl) and incubated for 3 min. After that, 50 µl of 10% Na_2_CO_3_ *w*/*v* were added to the mixture and samples were left in dark at RT for 60 min, mixing every 10 min. The absorbance was read at 785 nm against a blank consisting of deionized water instead of sample by using an UV-Vis spectrophotometer (Shimadzu UV 1601, Kyoto, Japan). Results were expressed as milligrams of gallic acid equivalents (GAE)/100 g of CLJ.

### Total Flavonoids Content

Total flavonoids were determined according to Bazzicalupo et al. (2019) with some modifications. Briefly, 450 µl of deionized water were added with 50 µl of sample (6.33–50.6 µl/6.25–50 mg/ml of CLJ diluted in deionized water) or rutin as reference compound (125–1000 µg/ml), mixed with 30 µl of 5% NaNO_2_ and incubated for 5 min. After that, 60 µl of 10% AlCl_3_ were added to the mixture and incubated for 6 min. Then, 0.2 mL of sodium hydroxide (1 M) and 210 µl of deionized water were added and the absorbance was recorded at 510 nm against a blank consisting of deionized water instead of sample by an UV-Vis spectrophotometer (Shimadzu UV-1601, Kyoto, Japan). Results were expressed as milligrams of rutin equivalents (RE)/100 g of CLJ.

### Ascorbic Acid Determination

The determination of ascorbic acid content was carried out according to Mazurek and Jamroz (2015) with some modifications. A sample solution consisting of CLJ and 2% *m*-phosphoric acid (1:1 *v*/*v*), shielded from light in order to prevent the loss of ascorbic acid, was filtered through a 0.22 µm nylon syringe filter and injected into a high performance liquid chromatography system equipped with a photodiode-array detector (HPLC-DAD, Agilent technologies, Santa Clara, CA, United States). The chromatographic separation was performed by a 5 µm Eclipse plus column (150 × 4.6 mm; Phenomenex, Torrance, CA, United States) maintained at 25°C using 0.025% trifluoroacetic acid solution as mobile phase. Flow rate and injection volume were 0.8 ml/min and 20 µl, respectively. UV-Vis spectra were acquired in the range 190–400 nm, whereas the acquisition of chromatograms was carried out at 254 nm. The peak identity was confirmed by comparing the retention time and UV–Vis spectra of sample with pure (≥99%) commercially available ascorbic acid standard. Quantification was carried out by an external standard calibration curve (concentration range 0.625–5 µg/ml).

### Polyphenols Characterization

The polyphenols characterization was carried out by a reversed-phase liquid chromatography coupled with diode array detection and electrospray ion trap mass spectrometry (RP-LC-DAD-ESI-MS) analysis. At this purpose a Luna Omega PS C18 column (150 × 2.1 mm, 5 µm; Phenomenex, Torrance, CA, United States) at room temperature (RT) and with a flow rate of 0.4 mL/min was used. The mobile phase and elution program was the same reported in Smeriglio et al. (2018b). The injection volume was 5 µl. The UV-Vis spectra were recorded in the 190–600 nm range and chromatograms were acquired at different wavelength (260, 292, 330 and 370 nm) to identified all polyphenol classes. The experimental parameters of the mass spectrometer (ion trap, Agilent model 6320) operating in both positive (ESI+) and negative (ESI−) ionization mode were set as follows: the capillary voltage was 3.5 kV, the nebulizer (N_2_) pressure was 40 psi, the drying gas temperature was 350°C, the drying gas flow was 9 L/min and the skimmer voltage was 40 V. The mass spectrometer was operated in full-scan mode in the m/z range 90–1000. Data were acquired by Agilent ChemStation version B.01.03 and Software Trap control version 6.2.

### *In Vitro* Cell-free Assays

#### Antioxidant Activity

The antioxidant activity of CLJ was evaluated by several *in vitro* assays, based on different mechanisms (electron, hydrogen or electron and hydrogen transfer-based assays) and reaction environments. Results, obtained by three independent experiments in triplicate (*n* = 3) were expressed as inhibition percentage (%) of the oxidative/radical activity, calculating the half-maximal inhibitory concentration (IC_50_) with the respective confident limits (C.L.) at 95%. All concentration ranges following reported refer to final volumes/concentrations of CLJ and reference compounds in the reaction mixture.

##### DPPH Assay

The scavenging activity against the DPPH radical was evaluated according to Smeriglio et al. (2017). Briefly, 37.5 µl of CLJ diluted in deionized water (0.030–0.243 µl/30.0–240.0 µg/ml) or trolox as reference compound (0.625–5.0 µg/ml) were added to fresh 10^–4^ M DPPH methanol solution (1.5 ml), mixed and incubated in the dark for 20 min. The decrease in absorbance was recorded at 517 nm against a blank consisting of deionized water instead of sample by using an UV–Vis spectrophotometer (Shimadzu UV-1601, Kyoto, Japan).

##### Trolox Equivalent Antioxidant Capacity (TEAC)

The scavenging activity against the ABTS radical was evaluated according to Monforte et al. (2018). Briefly, the radical solution was prepared by mixing 4.3 mM K_2_S_2_O_8_ and 1.7 mM ABTS (1:5, *v/v*) and incubating for 12 hours in dark at RT. The reaction mixture was then diluted with deionized water (absorbance of 0.7 ± 0.02) and used within 4 hours. Fifty microliters of CLJ diluted in deionized water (0.005–0.040 µl/5.0–40.0 µg/ml) or trolox as reference compound (0.625–5.0 µg/ml) were added to 1 ml of the reagent and incubated at RT for 6 min. The absorbance was recorded at 734 nm against a blank consisting of deionized water instead of sample by an UV-Vis spectrophotometer (Shimadzu UV-1601, Kyoto, Japan).

##### Ferric Reducing Antioxidant Power (FRAP)

The FRAP assay was carried out according to Smeriglio et al. (2019b). Fifty microliters of CLJ diluted in deionized water (0.08–0.65 µl/80.0–640.0 µg/ml) or trolox as reference compound (1.25–10.0 μg/ml) were added to freshly and pre-warmed (37°C) working FRAP reagent (1.5 ml). After 4 min of incubation at RT in the dark, the absorbance was recorded at 593 nm by an UV-Vis spectrophotometer (Shimadzu UV-1601, Kyoto, Japan) against a blank consisting of deionized water instead of sample.

##### Oxygen Radical Absorbance Capacity (ORAC)

The ORAC assay was carried out according to Smeriglio et al. (2019c). Briefly, 20 µl of several CLJ dilutions (0.002–0.016 µl/2.0–16.0 µg/ml), trolox as reference compound (0.25–2.5 µg/ml) or deionized water as blank were added to 120 µl of 117 nM fluorescein solution. After an incubation time of 15 min at 37°C, 60 µl of 40 mM AAPH were added to each well starting the reaction, which was monitored every 30 s for 90 min (λ_ex_ 485 nm; λ_em_ 520 nm) by a fluorescence plate reader (FLUOstar Omega, BMG LABTECH, Ortenberg, Germany).

##### Iron-Chelating Activity

The ferrozine assay was carried out according to Smeriglio et al. (2019c). Fifty microliters of 2 mM FeCl_2_·4H_2_O solution were added in 100 µl of CLJ sample diluted in deionized water (0.05–0.40 µl/50.0–400.0 µg/ml), EDTA (1.5–12 µg/ml) as reference compound or deionized water as blank and incubated at RT for 5 min. Then, 100 µl of 5 mM ferrozine solution and 3 ml of deionized water were added to the mixture and incubated for 10 min at RT. The absorbance was read at 562 nm by an UV-Vis spectrophotometer (Shimadzu UV-1601, Kyoto, Japan).

##### β-Carotene Bleaching

The test was carried out using an emulsion prepared according to Barreca et al. (2016). Eight milliliters of fresh emulsion were mixed with 0.23 µl of CLJ samples diluted in deionized water (0.10–0.81 µl/100.0–800.0 µg/ml), BHT (0.031–0.25 µg/ml) as reference compound or deionized water as blank. A β-carotene-free emulsion was used as negative control. The mixture was initially measured at 470 nm (*t = 0*), by an UV-Vis spectrophotometer (Shimadzu UV-1601, Kyoto, Japan) and then incubated in a water bath (50°C) for 120 min, recording the absorbance every 20 min.

#### Anti-Inflammatory Activities

##### Bovine Serum Albumin Denaturation Assay

This assay, which evaluates the inhibitory activity of CLJ on BSA-heath induced-denaturation, was carried out according to Saso et al. (2001). Briefly, 100 µl of 0.4% fatty free BSA solution and 20 µl of phosphate buffered saline (PBS, pH 5.3) were added to 80 µl of CLJ samples diluted in deionized water (0.40–3.24 µl/0.40–3.20 mg/ml). The solution was read initially (*t =* 0) and after an incubation time of 30 min (*t* = 30) at 70°C, recording the absorbance at 595 nm by a plate reader (Multiskan GO; Thermo Scientific, MA, United States). A blank with deionized water instead of sample was used as negative control. The anti-inflammatory power was expressed as inhibition (%) of albumin denaturation (ID), and it was calculated as follows:

$$\%\;ID=\frac{\left(1-(A-B)\right)}{\left(C-B\right)})\times 100$$

where A = sample absorbance at *t = 30;* B = blank absorbance at *t = 0; C =* blank absorbance at *t = 30.*

The IC_50_ with the respective C.L. at 95% was calculated.

##### Anti-protease Activity

The anti-tryptic activity of CLJ was evaluated according to Oyedapo and Famurewa (1995). Briefly, 200 µl of several CLJ samples diluted in deionized water (0.06–0.51 µl/63.0–500.0 µg/ml) were added to the reaction mixture consisting of 12 µl of trypsin (10 µg/ml) and 188 µl of 25mM Tris-HCl buffer (pH 7.5), both diluted in deionized water. After that, 200 µl of 0.8% casein were added and the reaction mixture was incubated for 20 min at 37°C in a water bath. At the end of the incubation time, 400 µl of perchloric acid were added to stop the reaction and to allow the protein precipitation. The cloudy suspension was centrifuged at 3500 × g for 10 min and the absorbance of the supernatant was recorded at 280 nm against a blank consisting of deionized water instead of sample, using an UV-Vis spectrophotometer (Shimadzu UV-1601, Kyoto, Japan). Diclofenac sodium (20.0–80.0 µg/ml) was used as reference compound. Results, obtained by three independent experiments in triplicate (*n* = 3) were expressed as inhibition percentage (%) of the protease activity, calculating the IC_50_ with the respective C.L. at 95%.

### *In Vitro* Cell-Based Assays

Overnight fasting (12 hours) venous blood samples from 10 healthy donors chosen according to specific inclusion (signed informed consent, males and females, aged between 23 and 40 years, non-smokers) and exclusion criteria (history of drug abuse including alcohol, participation in experimental trials within 3 months prior to study, use of any kind of drugs within the previous 3 months, pathologies) were collected in heparinized tubes.

#### Preparation of Erythrocytes Suspension

Erythrocytes suspension was prepared according to Gunathilake et al. (2018). Briefly, the fresh blood samples were centrifuged at 2400 × g for 5 min and the erythrocytes layer washed three times 1:1 (*v*/*v*) with saline solution (0.9% NaCl). After the centrifugation, the packed erythrocytes were suspended at 10 and 1% (*v*/*v*) with 10 mM sodium phosphate buffer pH 7.4 (PBS) for heat-induced hemolysis and free radical scavenging activity against intracellular reactive oxygen species (ROS) assays, respectively.

##### Heat-Induced Hemolysis Assay

According to Gunathilake et al. (2018), 0.05 ml of 10% erythrocytes suspension and 0.05 ml of several CLJ samples diluted in PBS (0.21–0.82 µl/200.0–800.0 µg/ml), diclofenac sodium 50–200 µg/ml (positive control) or phosphate buffer (negative control) were added to 2.95 ml of PBS. The mixture was incubated at 54°C for 20 min in a water bath and then centrifuged at 2000 × g for 3 min. The absorbance of the supernatant was recorded at 540 nm using an UV-Vis Spectrophotometer (Shimadzu UV-1601, Kyoto, Japan) and the level of hemolysis was calculated using the following equation:

% hemolysis inhibition = 100 x (1 – As/Ac)

where *As* is the absorbance of the test sample and *Ac* is the absorbance of the negative control.

##### Free-Radical Scavenging Activity Against Intracellular ROS

The free-radical scavenging activity of CLJ against the intracellular ROS was evaluated according to Wìden et al. (2012) with some modifications. Briefly, 0.01 ml of CLJ samples diluted in PBS (0.03–0.10 µl/25.0–100.0 µg/ml), trolox 12.5–50 µg/ml (positive control) or PBS (negative control) were added to 1 ml of 1% erythrocytes suspension, mixed and incubated in the dark for 2 h at 20°C under constant agitation (120 rpm) using an Innova 4000 benchtop incubator shaker (New Brunswick Scientific, NJ, United States). The erythrocytes were washed three times in PBS 1:1 (*v*/*v*) and centrifuged at 2400 × g for 5 min in order to remove any extracellular trace of antioxidant compounds. The cell pellet was lysed by the addition of distilled water (0.1 ml) and 1 ml of DCF-DA working solution in PBS (0.28 µg/ml) was added. After this, 18.9 µl of 30% H_2_O_2_ (167 mM) was added and the degree of ROS release monitored by recording the fluorescence intensity after 10 min (λ_ex_ 485; λ_em_ 535).

#### Peripheral Blood Mononuclear Cell (PBMC) Isolation

PBMC isolation was carried out according to Viji and Helen (2008). Briefly, Histopaque 1.077 was placed in a 15 ml tube and whole blood was stratified on the top. After the centrifugation (400 × g for 30 min at 20°C, with 9/3 acceleration/deceleration ramp), the PBMC were carefully recovered from the buffy coat and washed three times with PBS. The pellet was resuspended in culture medium consisting of RPMI 1640 supplemented with 2 mM L-glutamine, 10% FBS and 1% penicillin/streptomycin and cell concentration brought to 1 × 10^6^ cells/ml. Cell viability was assessed by trypan blue staining under the microscope.

##### Anti-inflammatory Activity

The anti-inflammatory activity of CLJ was evaluated according to Hougee et al. (2005) with some modifications. Briefly, freshly PBMC suspension (150 µl) were seeded in a 96-wells cell culture plate (Nunc^®^, Merck, Darmstadt, Germany). Twenty microliters of CLJ samples diluted in PBS (0.26–1.03 µl/0.25–1.0 mg/ml), diclofenac sodium 12.5–50 µg/ml (positive control) or PBS (negative control) were added and cells were incubated for 1 h at 37°C, 5% CO_2_. After that, inflammation was induced by adding 30 µl of LPS (10 ng/ml) in each well, incubating for another 16 h at 37°C, 5% CO_2_.

Cell supernatants were harvested and interleukin-6 (IL-6) and tumor necrosis factor α (TNF-α) release was measured by high-sensitivity human ELISA kits (DRG Diagnostics GmbH, Marburg, Germany) according to the manufacturer's recommendations.

### *In Vivo* Evaluation of the Anti-angiogenic Properties

#### Chick Chorioallantoic Membrane (CAM) Assay

The evaluation of the anti-angiogenic properties of CLJ was carried out according to Smeriglio et al. (2019d). Briefly, fertilized eggs of *Gallus gallus* were incubated for 4 days in a humidified incubator at 37°C, in order to favour the vessels growth and the CAM development. Eggs, which showed malformed or dead embryos were discarded. The CLJ samples diluted in deionized water (0.09–0.71 µl/90.0–700.0 µg/ml), retinoic acid (10 µg/ml) as positive control or deionized water as negative control were applied directly on the CAM surfaces and eggs were incubated for 24 h. The anti-angiogenic effect was evaluated by counting the blood vessel branch points in a standardized area by a stereomicroscope (SMZ-171 Series, Motic, Hong Kong, China) equipped with a digital camera (Moticam^®^ 5 plus). Images were processed by the GNU Image Manipulation Program (GIMP version 2.10.2).

Results, obtained by three independent experiments in quintuplicate (*n* = 5) were expressed as inhibition percentage (%) of the angiogenic activity, calculating the IC_50_ with the respective C.L. at 95%.

#### Endogenous Alkaline Phosphatase (EAP) Activity on Zebrafish Embryos

The quantification of EAP in zebrafish (*Danio rerio*, Hamilton) embryos was carried out according to Iannuzzi et al. (2019) following the ethical guidelines described by National Institute of Health Guide for Care and Use of Laboratory Animals.

Briefly, several male and female zebrafish specimens were purchased by a local pet store and kept in an aquarium at 28°C with 14/10 (light/dark) photoperiod. After a natural mating, embryos were generated and cultured in water at 28.5°C. Twenty-four hours post-fertilization (hpf), the healthy and regular ones were selected. After dechorionizing, embryos were distributed in a 96-well plate and incubated with 100 µl of embryos water containing different volumes/concentrations of CLJ (0.13–0.51 µl/125.0–500.0 µg/ml) or reference standard 2-methoxyestradiol (2-ME, 30 µg/ml). Deionized water was used as negative control. All embryos were incubated for 72 hpf. After that, embryos were dehydrated by using increasing concentrations of ethanol, washed three time with 1 M diethanolamine buffer (pH 9.8) and then incubated for 30 min at RT with *p-*nitrophenyl phosphate disodium salt (substrate). At the end of incubation time, 2 M NaOH was added to stop the reaction. The absorbance of soluble EAP was recorded at 405 nm by a microplate reader (Multiskan GO; Thermo Scientific, MA, United States).

Results, obtained by three independent experiments in quintuplicate (*n* = 5), were expressed as inhibition percentage (%) of the EAP activity, calculating the IC_50_ with the respective C.L. at 95%.

### Statistical Analysis

Results were expressed as the average ± standard deviation (S.D.) of three independent experiments in triplicate (*n* = 3) for *in vitro* cell-free and cell-based experiments and of three independent experiments in quintuplicate (*n* = 5) for *in vivo* experiments. The statistical significance was evaluated by one-way analysis of variance (ANOVA) followed by Tukey’s test using SigmaPlot 12.0 software. Data were considered statistically significant for *p* < 0.05.

## Results

### Phytochemical Analyses

**FIGURE 1:**
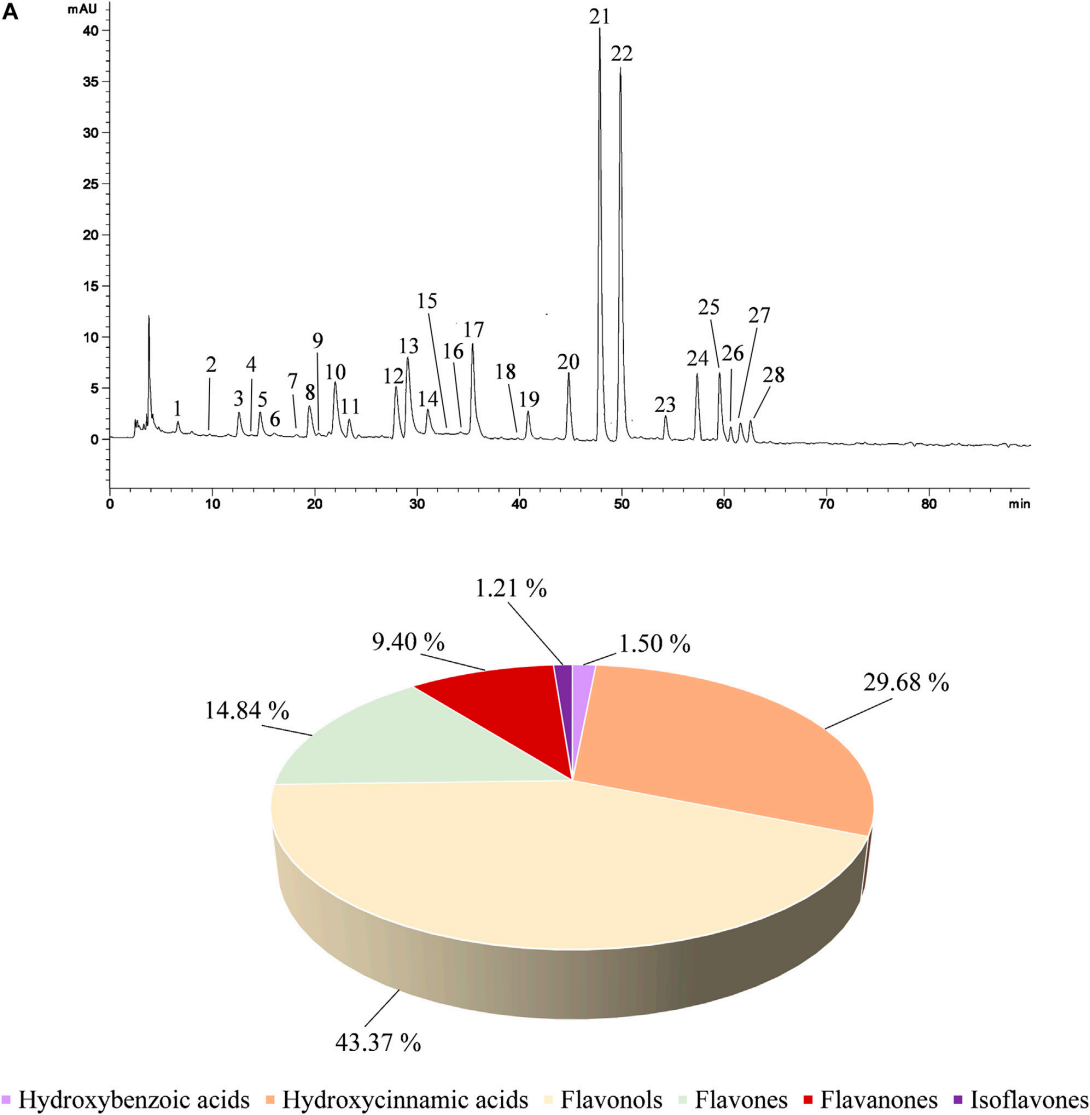
Representative LC-DAD chromatogram of CLJ acquired at 330 nm **(A)**. Peak numbers refer to compounds listed in Table 1 according to their elution order. **Panel B** reports the composition of CLJ in terms of polyphenols classes expressed as mean area percentage with respect to all polyphenols identified.

This study investigates for the first time the polyphenol profile and the biological activity of CLJ. Preliminary phytochemical screening using *in vitro* colorimetric tests revealed a high content of total phenols (819.05 ± 37.68 mg GAE/100 g of CLJ) as well as a very high content of flavonoids (1054.40 mg RE/100 g of CLJ). These preliminary data were confirmed by the determination of the polyphenol profile by RP-LC-DAD-ESI-MS analysis, which allowed to identify 28 polyphenols belonging to 6 different classes. The predominant class is that of flavonols (43.37%) followed by hydroxycinnamic acids (29.68%), flavones (14.84%), flavanones (9.40%), hydroxybenzoic acids (1.50%) and isoflavones (0.15%) (Figure 1; Table 1).

**TABLE 1:** Characterization of the polyphenol profile of *C. lumia* juice by RP-LC-DAD-ESI-MS analysis.

Peak no.	Compound	RT	Area %	λ_max_	*m/z*	[M-H]^-^/[M-H]^+^
*Hydroxybenzoic acids*						
5	1,1′-biphenyl, 4-Hydroxybenzoic acid	14.70	1.50 ± 0.08	306	292	/293
*Hydroxycinnamic acids*						
2	Feruloyl-*O*-sinapoyl-*O*-caffeoylquinic acid	9.61	0.07 ± 0.00	280	736	735/737
3	(E)-3-(3,4-diacetoxy-5-methoxyphenyl)-acroyl-4-*O*-*p*-Coumaroyl-5-*O*-Caffeoylquinic acid	12.59	1.69 ± 0.02	302	778	777/
7	Caffeoylquinate shikmate derivative	18.14	0.13 ± 0.01	270	510	509/511
18	1-Sinapoyl-2-feruloylgentiobiose	39.74	1.31 ± 0.06	282; 350	724	723/725
22	1-Caffeoyl-5-feruloylquinic acid	49.80	**25.70 ± 1.55**	284; 336	530	531/529
26	3,4-Dicaffeoylquinic acid	60.66	0.78 ± 0.01	280; 336	516	515/
*Flavonols*						
4	Kaempferol 3,7,4′-*O*-triglucoside	13.79	0.07 **±** 0.00	304	772	771/773
8	Limocitrol-*O*-glucoside	18.79	0.23 ± 0.00	286; 328	538	537/539
17	Quercetin 3-(2''-*p*-hydroxybenzoyl-4''-*p*-Coumarylrhamnoside)	35.47	**6.77 ± 0.15**	290; 330	714	713/
19	Quercetin 3-*O*-(6"-malonyl-glucoside)-7-*O*-glucoside	40.62	0.11 ± 0.01	282; 350	712	711/713
21	Kaempferol 3′- rhamnoside	47.80	**23.12 ± 0.84**	272; 348	432	431/432
24	Quercetin 3-*O*-(6"-malonyl-glucoside)	57.68	**7.72 ± 0.38**	280; 336	550	549/551
25	Dihydroquercetin 3-*O*-rhamnoside	59.54	**4.41 ± 0.18**	284; 316	449	448/450
28	Isorhamnetin 3(7)-*O*-glucuronopyranosyl-(1-2)-*O*-glucuronopyranoside	62.62	0.94 ± 0.04	284; 326	668	669/667
*Flavones*						
1	Apigenin 7-*O*-glucuronide	6.61	0.57 ± 0.02	280; 300	446	445/447
9	Apigenina-7-*O*-glucoside	19.47	1.56 ± 0.03	280; 328	432	431/433
6	Myricetin 3-*O*-galactoside	15.83	0.08 ± 0.00	274	480	479/481
13	Diosmetin-6-*C*-b-*D*-glucoside	29.43	**9.35 ± 0.25**	260; 300	462	461/463
14	Diosmetin 6,8-di-*C*-glucoside	30.85	2.04 ± 0.08	260; 300	624	623/625
23	Tricin 7-*O*-[glucuronopyranosyl-(1-2)-*O*-methyloglucuronopyranoside]	54.54	0.10 ± 0.01	276; 336; 370	696	695/697
27	Orientin-sulphate	61.56	1.14 ± 0.07	280; 336; 370	528	527/529
*Flavanones*						
10	Neohesperidin dihydrochalcone	21.96	**4.51 ± 0.08**	260; 296	612	611/613
11	Naringenin-7-*O*-glucoside	23.34	1.19 ± 0.03	284; 325; 370	433	432/434
15	Neoeriocitrin	32.89	0.05 ± 0.00	286; 326	596	595/597
16	Neohesperidin	34.35	1.68 ± 0.06	274; 334	610	609/611
12	5,7-dihydroxy-49-methoxyflavanone 7-*O*-Neohesperidoside	28.57	1.97 ± 0.03	280; 330	594	593/595
*Isoflavones*						
20	6''-*O*-Malonyl daidzin	45.81	1.21 ± 0.05	280; 338	502	501/503

Results were expressed as mean area percentage ± standard deviation (*n* = 3) of each compound with respect to the total area of polyphenols identified. Bold numbers refer to the most abundant compounds.

Table 1 shows the identified polyphenols classified according to the class they belong to and their relative abundance (mean area percentage) at 330 nm, wavelength at which it is possible to detect all compounds identified. The peak numbers refer to the elution order as shown in Figure 1. As can be seen from the representative chromatogram shown in Figure 1, the method used allowed to separate all compounds without any base-line interference or co-elution.

Since CLJ is an aqueous matrix, the polyphenol profile is mostly made up of glycosylated derivatives of both phenolic acids and flavonoids. The predominant compound is 1-caffeoyl-5-feruloylquinic acid belonging to the class of hydroxycinnamic acids, which represents the 25.70% of the identified polyphenols. Among the flavonoids, the predominant compound is kaempferol 3′-rhamnoside (23.12%) followed by diosmetin-6-*C*-b-*D*-glucoside (9.35%), quercetin 3-*O*-(6“-malonyl-glucoside) (7.72%), quercetin 3-(2''-*p*-hydroxybenzoyl-4''-*p*-coumarylrhamnoside) (6.77%), neohesperidin dihydrochalcone (4.51%) and dihydroquercetin 3-*O*-rhamnoside (4.41%) (Table 1).

Since *Citrus* juices are generally rich in vitamin C, the ascorbic acid content was determined by HPLC-DAD analysis, revealing that CLJ is a rich source of this metabolite (352 mg/kg of CLJ) that is well known as well as polyphenols for its marked anti-oxidant, anti-inflammatory and anti-angiogenic properties.

### Antioxidant and Anti-inflammatory Properties

**FIGURE 2:**
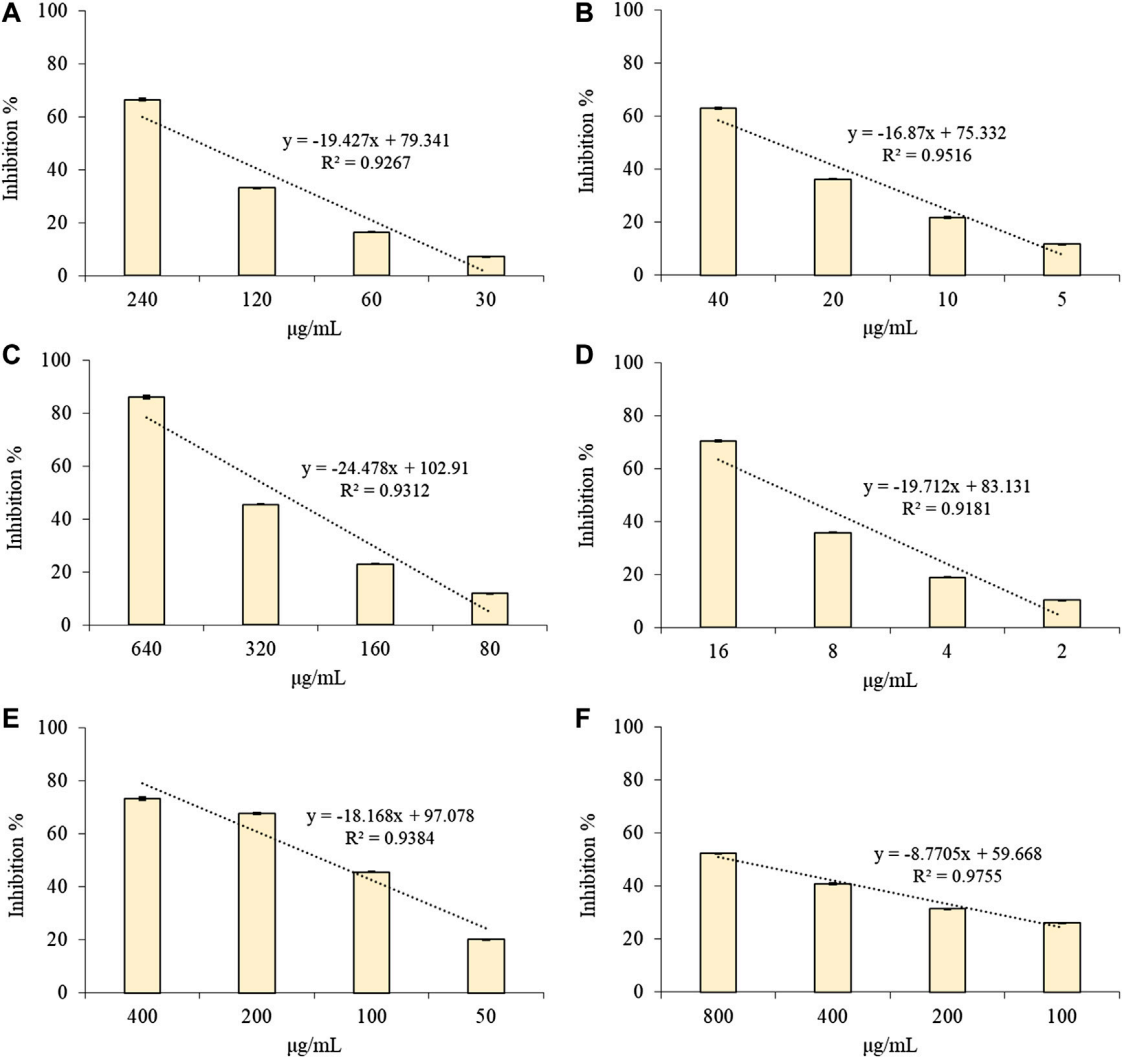
Antioxidant and free radical-scavenging activity of several concentrations of CLJ towards DPPH **(A)**; TEAC **(B)**; FRAP **(C)**; ORAC **(D)**; Iron-chelating activity **(E)** and *β*-carotene bleaching **(F)** assay. Results were expressed as mean inhibition percentage ± standard deviation of three independent experiments (*n* = 3).

The antioxidant and free-radical scavenging properties of CLJ were tested firstly by several *in vitro* colorimetric assays based on different environments and reaction mechanisms as well as against differently charged radicals. CLJ showed a strong and dose-dependent (*R* ^2^≥ 0.9181) antioxidant and free radical-scavenging activity with the following order of potency: ORAC > TEAC > β-carotene bleaching > DPPH > FRAP > Iron-chelating activity (Figure 2).

Table 2 shows the IC_50_ values (µg/ml) of CLJ in comparison with those of the reference standards trolox and BHT.

**TABLE 2:** *In vitro* antioxidant and anti-inflammatory activity of *C. lumia* juice (CLJ) in comparison with reference compounds.

Assay	CLJ	Reference compound*
Antioxidant activity		
TEAC	26.26 (21.28–32.65)^*^	2.92 (1.82–4.40)
ORAC	10.22 (8.47–12.19)^*^	0.67 (0.33–1.22)
β-carotene bleaching	70.80 (47.06–106.71)^*^	0.21 (0.11–0.36)
Iron-chelating activity	286.20 (237.11–345.15)^*^	6.58 (5.20–8.08)
FRAP	260.11 (142.62–496.165)^*^	3.75 (1.71–7.59)
DPPH	160.81 (137.04–194.26)^*^	3.87 (1.18–5.45)
Anti-inflammatory activity		
BSA denaturation assay	1457.10 (1242.32–1709.25)*	39.21 (32.09–47.89)
Anti-protease activity	253.22 (186.02–345.15)*	33.48 (28.29–39.63)

^*^Trolox for TEAC, ORAC, FRAP and DPPH assays, BHT for β-carotene bleaching assay, EDTA for iron-chelating activity. Diclofenac sodium for bovine serum albumin denaturation assay and anti-protease activity. ^*^ *p* < 0.01. Results were expressed as half-inhibitory concentration (IC_50_ µg/ml) with confident limits (C.L.) at 95%.

Despite there is a statistically significant difference (*p* < 0.01) in comparison with the reference compounds, it is necessary to underline that CLJ has been tested simply diluted in deionized water, therefore we are talking about a food as it is, and not of a polyphenol extract. Considering this, CLJ has proven to be a plant complex with a very strong antioxidant and free-radical scavenging activity, because these activities are translatable, *in vitro*, considering the density of the juice reported in the *Total Phenols Content*, to a few microliters of fresh juice (∼0.002–0.243 µl).

Preliminary screening of the anti-inflammatory activity of a plant complex can be evaluated *in vitro* by two simple colorimetric assays, the BSA denaturation assay and the anti-protease activity test.

**FIGURE 3:**
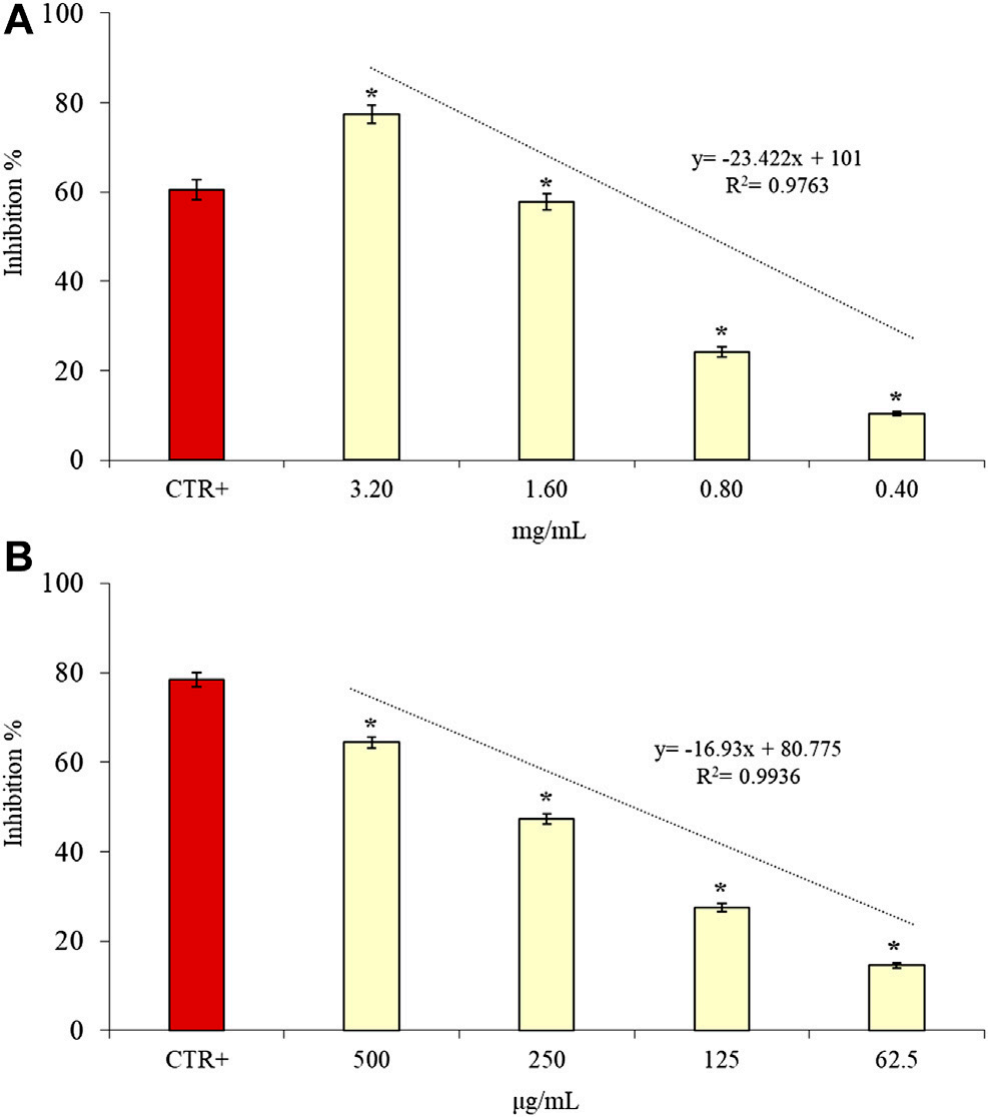
Anti-inflammatory activity of several concentrations of CLJ evaluated by BSA denaturation **(A)** and anti-protease activity **(B)** assays. Diclofenac sodium (50 and 80 µg/ml, respectively) was used as reference compound in both assays. Results were expressed as mean inhibition percentage ± standard deviation of three independent experiments (*n* = 3). *p < 0.01 *vs* positive control (CTR+).

Anti-inflammatory drugs act by protecting endogenous proteins against denaturation (Saso et al., 2001). At this purpose, the effect of CLJ on heat-induced BSA denaturation was evaluated in comparison with the anti-inflammatory drug diclofenac sodium as positive control (Figure 3A).

CLJ showed a strong anti-inflammatory activity by inhibiting, in statistically significant (*p* < 0.01) and dose-dependent manner (*R* ^2^ = 0.9763), the heat-induced BSA denaturation. Moreover, the highest concentration tested (3.20 mg/ml equal to ∼3 µl of CLJ) showed the best anti-inflammatory activity (77.35%) in comparison with the positive control diclofenac sodium 50 µg/ml (60.51%).

The anti-tryptic activity of CLJ was investigated, as anti-inflammatory marker, considering that proteases have been implicated in several inflammatory-based diseases promoting the tumorigenesis and invasive growth (Oyedapo and Famurewa, 1995).

CLJ showed, also in this case, a strong anti-inflammatory activity (64.41% at the highest concentration corresponding to ∼0.5 µl of CLJ) with a statistically significant (*p* < 0.01) and dose-dependent behavior (*R* ^2^ = 0.9936) with respect to the positive control diclofenac sodium that, at the highest concentration tested (80 µg/ml), showed an anti-tryptic activity of 78.48% (Figure 3B).

The IC_50_ values with the respective C.L. of CLJ in comparison with the positive control (diclofenac sodium) were reported for both anti-inflammatory assays in Table 2.

After this preliminary screening of the antioxidant and anti-inflammatory activity by cell-free tests, cell-based assays were caried out to deepen investigate the CLJ behavior, which did not interfere, also as pure juice, with the cell viability (data not shown).

The antioxidant results were confirmed by the erythrocytes-based assay, which allowed us to investigate the free-radical scavenging activity of CLJ against the H_2_O_2_-induced intracellular ROS.

**FIGURE 4:**
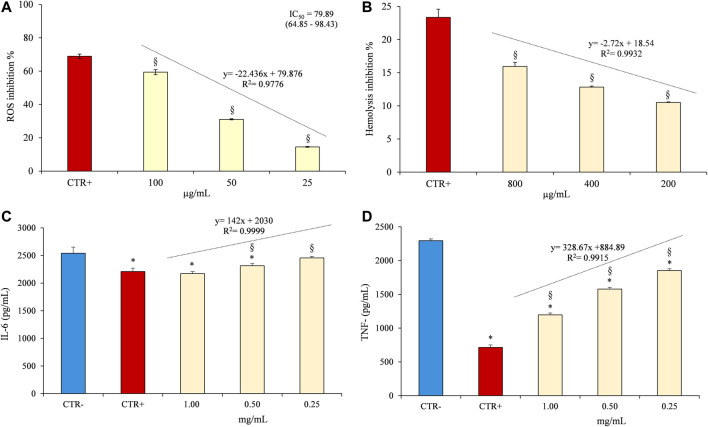
Evaluation of antioxidant and anti-inflammatory activity of CLJ by cell-based assays carried out on erythrocytes **(A,B)** and PBMC **(C,D)**. **(A)** Heat-induced hemolysis, diclofenac sodium 50 µg/ml was used as positive control (CTR+); **(B)** Antioxidant activity against intracellular ROS, trolox 50 µg/ml was used as positive control (CTR+); **(C,D)** IL-6 and TNF-α release by PBMC after LPS-induced inflammation, diclofenac sodium 50 µg/ml was used as positive control (CTR+) whereas cell medium culture containing 0.1% DMSO was used as negative control (CTR-). *p < 0.001 *vs* CTR-; ^§^ *p* < 0.001 *vs* CTR+.

In fact, as can be seen from Figure 4A, CLJ showed, also in this case, a strong and dose-dependent (*R* ^2^ = 0.9776) antioxidant activity (inhibition % = 14.53–59.40) with an IC_50_ value equal to 79.78 µg/ml (corresponding to ∼0.08 µl of CLJ). As previously observed in the cell-free assays, a statistically significant difference (*p* < 0.01) was found between the behavior of CLJ and the positive control (trolox). However, the reference compound showed, at a concentration equal to 50 µg/ml, an inhibition of the H_2_O_2_-induced intracellular ROS of 68.92%, with an IC_50_ equal to about half of the CLJ (34.56 µg/ml, C.L. 28.69–41.62), flattening the difference found in cell-free tests and validating further the antioxidant and free-radical scavenging power of CLJ.

The anti-inflammatory activity of CLJ was evaluated firstly as erythrocyte membrane stabilizer in the heat-induced hemolysis test and then monitoring the IL-6 and TNF-α release after LPS-induced inflammation on PBMC.

The inhibition percentages of the heat-induced hemolysis of red blood cells at different concentrations of CLJ (200–800 µg/ml) are shown in Figure 4B. Although CLJ shows, also in this case, a dose-dependent behavior (*R* ^2^ = 0.9932), it inhibits hemolysis, as pure juice, by a maximum of 15.95%, not allowing to calculate an IC_50_. However, it should be noted that also the positive control, diclofenac sodium, a well-known anti-inflammatory drug, shows a weak activity in this test, showing an IC_50_ equal to 128.74 µg/ml (C.L. 105.78–156.70).

The second experimental model based on PBMC allowed us to gain greater insight into the potential anti-inflammatory activity of CLJ, also corroborating what was previously observed with the cell-free tests. Figures 4C,D show the results of the IL-6 and TNF-α release, respectively after the LPS-induced inflammation on PBMC model. CLJ showed not only a dose-dependent behavior (*R* ^2^ = 0.9999) but it decreased significantly (*p* < 0.01) the IL-6 release by PBMC with respect to the negative control. Moreover, no statistically significant difference was found between the highest concentration of CLJ tested (1 mg/ml corresponding to ∼1 µl of CLJ) and the positive control (diclofenac sodium 50 µg/ml). The strong antioxidant activity of CLJ has been highlighted even more clearly by the analysis of the TNF-α release by PBMC (Figure 4D). CLJ decreased significantly (*p* < 0.01) the TNF-α release in comparison with the negative control at all concentrations tested (from 1.92 folds at the highest concentration to 1.24 folds at lowest concentration) with a dose-dependent behavior (*R* ^2^ = 0.9915).

These results, even if significantly different (*p* < 0.01) with respect to the diclofenac sodium 50 µg/ml, which showed the strongest inhibition of the TNF-α release (3.21 folds with respect to the negative control), show how CLJ, simply a food, is able to exert a strong anti-inflammatory activity even in a cellular model at very low concentrations, which correspond to very few microliters of juice.

### Anti-angiogenic Properties

**FIGURE 5:**
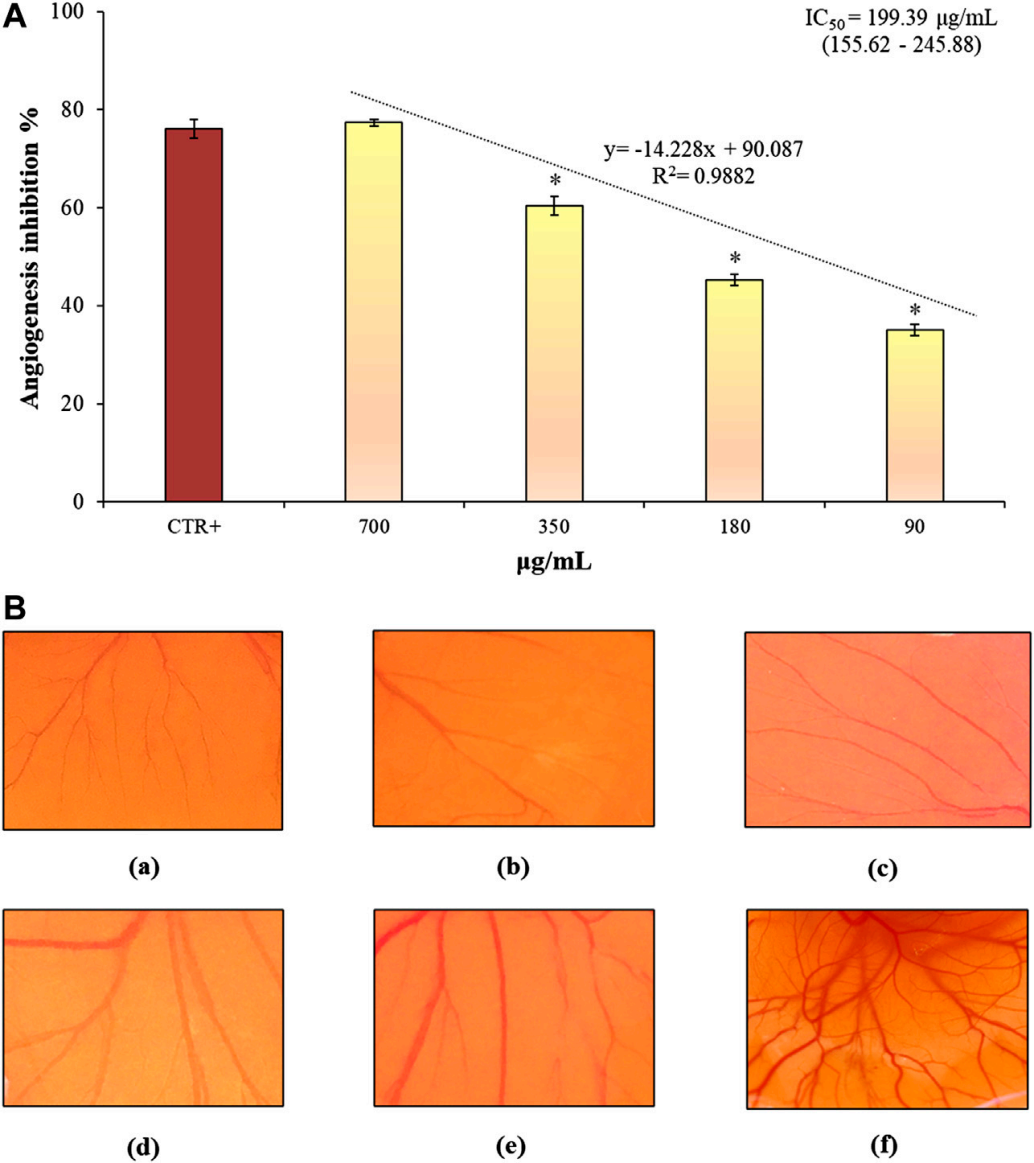
Anti-angiogenic properties of several concentrations of CLJ evaluated by CAM assay **(A)**. In **(B)**, representative microscopic images of the CAMs after treatment with the reference compound (retinoic acid, 10 µg/ml) **(A)**, 700–90 µg/ml CLJ samples **(B-E)** and negative control (deionized water) **(F)** are reported. Results were expressed as mean inhibition percentage ± standard deviation of three independent experiments (*n* = 5). *p < 0.01 *vs* positive control (CTR+) retinoic acid 10 µg/ml.

The CAM model was used as *in vivo* model to assess the effects of CLJ on angiogenesis. As it is reported in Figure 5A, the inhibitory effects on the neovascularization of the CAMs expressed as inhibition percentage (%) *vs* negative control (100% of neovascularization), ranged from 35.04 to 77.39% in the concentration range tested (90–700 µg/ml). The anti-angiogenic activity of the highest concentration of CLJ (77.39%) was comparable with that of the reference standard retinoic acid (76.19%). Indeed, no statistically significant difference was found between the highest concentration of CLJ (700 µg/ml) and the positive control (retinoic acid, 10 µg/ml) (Figure 5A). Conversely, the lower CLJ concentrations (90–350 µg/ml) showed a statistically significant difference with respect to the positive control, showing lowest and dose-dependent (*R* ^2^ = 0.9882) inhibitory activities (Figure 5A). The IC_50_ value of CLJ with the respective C.L. at 95% was also calculated highlighting a very interesting results (199.39 µg/ml, 155.62–255.47) considering, above all, that this concentration corresponds to a few microliters of pure CLJ (∼ 0.20 µl) (Figure 5A).

In Figure 5B representative microscopic images of the CAMs after treatment with the reference compound (retinoic acid, 10 µg/ml) (a), CLJ samples (b-e) and negative control (deionized water) (f) are reported. CAMs of control eggs showed the presence of a clear vascular network with large vessels (Figure 5F). Conversely, in the CAMs treated with both retinoic acid and CLJ sample (a-e), an evident decrease of the blood vessel branch points was found. Indeed, the vessels density is correlated to the blood supply to the standardized area taken into account, whereas it is inversely correlated to the inhibition degree.

*Danio rerio* known as zebrafish is an ideal experimental model to assess the angiogenic response of test samples and to identify any side effects including toxicity.

**FIGURE 6:**
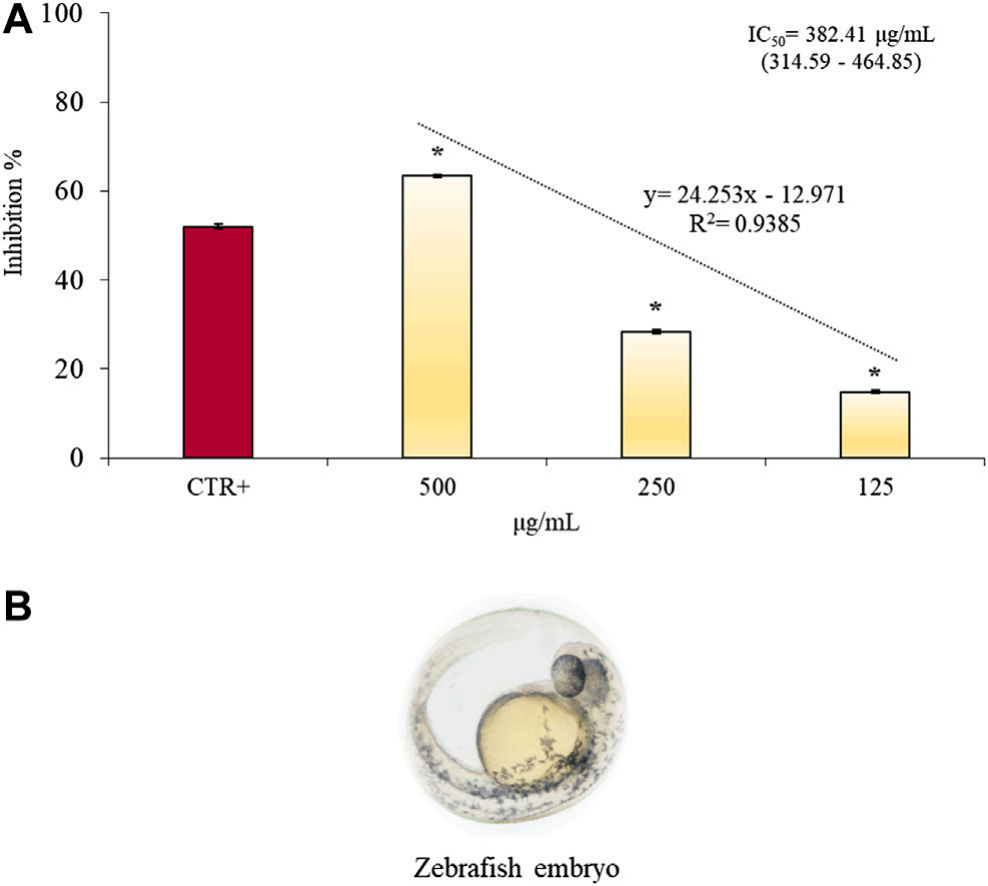
Anti-angiogenic properties of several concentrations of CLJ (125–500 µg/ml) evaluated by EAP activity of vascular endothelial cells released from treated zebrafish embryos **(A)**. **(B)** shows a microscopic image of Zebrafish embryo (magnification 20×). Results were expressed as mean inhibition percentage ± standard deviation of three independent experiments (*n* = 5). *p < 0.01 *vs* positive control (CTR+) 2-methylestradiol 30 µg/ml.

As a marker of vessel growth, the EAP activity of vascular endothelial cells released from treated embryos was quantified. Results of CLJ activity on EAP, expressed as inhibition percentage with respect to the negative control (100% of EAP activity), are reported in Figure 6.

CLJ samples (125–500 µg/ml corresponding to 0.13–0.51 µl) showed a strong and dose-dependent (*R* ^2^ = 0.9385) anti-angiogenic response reducing significantly (*p* < 0.01) the EAP activity in zebrafish embryos in comparison with the positive control (2-ME, 30 µg/ml). In particular the highest concentration of CLJ tested showed the strongest (63.37%) and statistically significant (*p* < 0.01) anti-angiogenic activity with respect to the reference compound 2-ME (52.0%) without altering survival or inducing malformation to the zebrafish embryos (data not shown).

## Discussions

This is the first study, which investigates the polyphenol profile, the ascorbic acid content as well as the antioxidant, anti-inflammatory and anti-angiogenic properties of *Citrus lumia* juice. However, several researches are available on the characterization and biological activities of the juices of different *Citrus* species, which allow to critically discuss what it was observed in the present study, also correlating it to the different classes of bioactive compounds identified.

*Citrus* juices, in particular the hand-squeezed (raw), are one of the main sources of nutrients as well as of secondary metabolites in a standard balanced diet. A part ascorbic acid, one of the main groups of compounds responsible for the health effects of *Citrus* juices are polyphenols with special reference to phenolic acids and flavonoids (Barreca et al., 2020). According to our results, flavanones and flavones as well as flavonols, which derived from the flavanones following hydroxylation in 3-position, are generally the most abundant classes of the flavonoid pattern of *Citrus* juices, occurring generally in their glycosylated forms (Barreca et al., 2020). As demonstrated in this study, the most common flavones identified in *Citrus* species are the glycosylated forms of luteolin, apigenin and diosmetin (Barreca et al., 2020). Flavones and flavanones showed remarkable antioxidant activity, not only acting as a free radical scavenger, but also indirectly modulating the cellular defenses via the NF-E2-related factor 2-antioxidant responsive element (Nrf2-ARE) pathway, which regulates the expression of several antioxidant genes such as heme oxygenase 1 (HO-1), glutathione peroxidase (GPX), and thioredoxin (TXN) (Barreca et al., 2017).

Moreover, they show promising anticancer activity because they are able to decrease the proliferative growth, modulate metabolic pathways and key enzymes (i.e. protein kinase, phosphodiesterase, lipoxygenase, cyclooxygenase, and phospholipase), arrest the cell cycle inducing apoptotic events, revert the multidrug resistance and inhibit the angiogenesis (Barreca et al., 2020).

Regarding flavonols, certainly the most investigated is quercetin. Quercetin and kaempferol derivatives represent the most abundant flavonols in CLJ.

Quercetin has several health properties such as blood pressure-lowering effects, antioxidant activity, inhibitory activity on angiotensin-converting enzyme (ACE), enhancing the endothelium function, decreasing the coronary heart disease incidence by attenuating the expression of metalloproteinase 1 and by interfering with the accumulation of atheromatous plaques (Shahidi and Yeo, 2018).

However, in light of our study, what is even more interesting is that quercetin suppress the cyclooxygenase-2 (COX-2) expression in human breast cancer cells by specifically targeting the p300 signaling pathway. COX-2 is an inducible enzyme, which plays a critical role in several patho-physiological events including inflammation, atherosclerosis, tissue injury, angiogenesis and tumorigenesis, up-regulating different pathways such as estimated glomerular filtration rate (eGFR), phosphoinositide 3-kinase (PI3k) and extracellular signal-regulated protein kinase (Erk1/2) signaling (Xiao et al., 2011).

Considering this, flavonoids play a pivotal role in the inhibition of the onset and development of inflammatory diseases.

Another class of polyphenols, which show remarkable antioxidant, anti-inflammatory and anti-carcinogenic activity are the phenolic acids.

Among them, hydroxycinnamic acids, according to our results, are the most abundant in plants. The main representatives are coumaric, caffeic, ferulic, and sinapic acids and their derivatives, although they are usually found as glycosylated derivatives or esters of quinic, shikimic and tartaric acids (Číž et al., 2020). The anti-carcinogenic potential of different phenolic acids has been investigated in several cell lines showing the ability of these compounds to influence the cell cycle, invasion and apoptotic behavior, inducing the expression of several tumor-suppressing proteins such as p53, phosphatase and tensin homolog (PTEN), p21, and p27. The anti-inflammatory activity of hydroxycinnamic acids is well known thanks to various studies carried out both *in vitro* and *in vivo*, by which it has been demonstrated that these compounds act predominantly by decreasing the expression of inflammatory mediators such as the IL-6, IL-1 and TNF-α (Shahidi and Yeo, 2018) as well as by decreasing the expression of nuclear factor kappa-light-chain-enhancer of activated B cells (NF-kB) p65, that recently has been correlated to the angiogenesis process by regulating key angiogenesis factors such as vascular endothelial growth factor (VEGF), transforming growth factor beta (TGF-β), IL-6, and TNF-α. Moreover, the inflammatory cytokines IL-6 and TNF-α are also well-recognized pro-angiogenic factors, which can accelerate the angiogenesis events inducing the vascular remodeling (Cao et al., 2016).

Moreover, it has been demonstrated that caffeic acid induces DNA oxidation of cancer cells and reduces tumor cell angiogenesis by blocking signal transducer and activator of transcription (STAT) proteins and suppressing the collagen IV metalloproteases MMP2 and MMP-9 (Espíndola et al., 2019).

However, a single bioactive compound, even if used at high concentrations, could not be sufficient in preventing or treating inflammatory diseases because several different pathways are involved in diseases progression (Cirmi et al., 2017). Therefore, the employment of plant complexes, sources of a wide range of bioactive compounds, which show additive and/or synergistic activity together with the simultaneous modulation of different intracellular pathways and cellular targets, represents the best choice.

One of the main issues against the clinical employment of polyphenols or plant complex rich in these bioactive compounds in anti-inflammatory therapy remains always their low bioavailability, although recent *in vivo* research and clinical trials show evidence to support their use (Hyson, 2015).

It has been demonstrated, for example, that pure (100%) fruit juice intake increases antioxidant status within 1 h after consumption, and that the effect may be sustained for several hours depending on the volume and type of juice, subject characteristics and several other unknown factors (Hyson, 2015). Remaining within the *Citrus* genus, orange juice exhibits a maximal antioxidant effect within 30 min of intake and induces a statistically significant decrease in ROS generation/oxidative stress in human plasma, sustained for 90 min. Moreover, since the *Citrus* fruits are very rich in ascorbic acid, the dietary intake of their juices was associated with an increase of the antioxidant status comparable to vitamin C supplementation (Hyson, 2015). Orange juice exerted antioxidant and anti-inflammatory properties at cellular and molecular level from 1 to 5 h after consumption and it was associated with a significant decrease of diastolic blood pressure compared to placebo and to hesperidin-enriched drink, suggesting a pivotal role for this flavanone in mediating vascular reactivity. Moreover, it has been observed that the intake of a mixed juice from three red orange varieties (Tarocco, Sanguinelllo, and Moro) in humans decrease significantly the C-reactive protein, IL-6, and TNF-α levels after 1 week of juice intake (Hyson, 2015).

A limited number of reports exists on the bioavailability of bioactive compounds in humans associated with *Citrus* juice intake. Franke et al. (2005) found that an intake of about 250 mL/day of fresh orange juice increases significantly the Vitamin C (+59%) and flavanones hesperetin and naringenin (512% and 874% respectively) concentrations after three weeks. The ascorbic acid content of CLJ (352 mg/kg) is close to that reported in the U.S. Department of Agriculture (USDA) food composition database (USDA, 2020b), with ascorbic acid content in *Citrus* raw juice, which vary between 300 and 500 mg/kg.

Ascorbic acid is well-known to alter the metabolic activity of endothelial cells by decreasing the ATP levels. This prevents cell proliferation and migration counteracting new blood vessel formation. However, this ability of ascorbic acid seems only secondary to the main mechanism involved into the anti-angiogenic effects of this molecule. Ascorbic acid is able to inhibit at high doses the nitric oxide (NO) release in endothelial cells, the key stimulus for new blood vessel formation, by changing the oxidative-reduction status within the cells and decreasing the NO availability through the formation of peroxynitrite (Mikirova et al., 2008).

As a result, a plant complex such as the *Citrus* juice, with a high content of phenolic acids, flavonoids and ascorbic acid with promising antioxidant, anti-inflammatory and anti-angiogenic properties could be an ideal candidate for the development of anti-cancer drugs (Ngoua-Meye-Misso et al., 2018).

The present study consisted of the phytochemical investigation, antioxidant, anti-inflammatory and anti-angiogenic activities of CLJ. In addition to the antioxidant effects, it showed a very strong anti-inflammatory activity compared to that of the reference drug, diclofenac sodium by inhibition of the protein denaturation (BSA) and of the protease activity. It is a well-known that denaturation of tissue proteins leads to inflammatory diseases and that the production of auto-antigens may be due to the denaturation of proteins *in vivo* (Ngoua-Meye-Misso et al., 2018).

Moreover, to deepen the antioxidant and anti-inflammatory activity of CLJ, two cell-based model were used (erythrocytes and PBMC). This allowed to investigate the interactions between CLJ and the functionally complete cellular enzyme patterns as well as cell membranes, providing more biological relevance to the present study. Erythrocytes are a relevant human cell model in the investigation of the antioxidant protection of natural compounds against oxidative stress as well as to test cell-permeating antioxidants, allowing an easy diffusion also of the glycosylated molecules by the glucose transport protein (GLUT1) (Widén et al., 2012). Moreover, the erythrocytes cell membrane is similar to the lysosomal one and its stabilization may delay or inhibit the lysis, counteracting the release of the cytoplasmatic content and decreasing consequently the tissue damage and the trigger of the inflammatory cascades (Gunathilake et al., 2018).

PBMC physiologically contribute to the tissue self-healing mechanisms. They are able to release cytokines that direct resident stem cells towards tissue regenerative processes and mainly perform three functions: (i) induce the formation of new blood vessels in the tissue to be regenerated (angiogenesis), (ii) modulate inflammation by inducing the reparative phase of the macrophage and (iii) can differentiate and intervene directly in the regenerative process (Beer et al., 2016).

During inflammation, they release IL-6 and TNF-α, pro-inflammatory cytokines, which play a pivotal role in different steps of several chronic inflammatory diseases. IL-6 regulates the growth and differentiation of various cell types that play a key role in the immune and hematopoietic system. It is also an important inducer of acute phase reactions in response to inflammation or tissue damage. TNF-α has anti-cancer and growth regulatory activities showing selectivity for tumor cells and carrying out pro-angiogenic activity. It also has immunomodulatory and pro-inflammatory activities by inducing the production of other inflammatory mediators such as IL-1, IL-6, prostaglandins, platelet activating factor (PAF), etc. (Muñoz and Costa, 2013).

CLJ showed a strong antioxidant and anti-inflammatory activity also in these two cell-based models, corroborating what observed in preliminary cell-free assays as well as the results of previous studies, which highlighted the strong antioxidant and anti-inflammatory activity of *Citrus* juices in inhibiting in particular the IL-6 and TNF-α release that, other than inflammatory markers, are recognized as key pro-angiogenic factors (Cao et al., 2016). This is even more true since the results of anti-inflammatory activity are also reflected in the anti-angiogenic activity observed *in vivo.* Indeed, CLJ showed strong and dose-dependent anti-angiogenic activity, by inhibiting the blood vessel formation on CAMs and by decreasing the EAP on zebrafish embryos. Moreover, within the concentration range tested, no cell viability alteration or dead or malformed embryos were recorded, indicating that the antioxidant, anti-inflammatory and anti-angiogenic effect observed was not due to the toxicity of CLJ.

## Conclusions

In conclusion, this study provides evidences that *C. lumia* juice have antioxidant, anti-inflammatory and anti-angiogenic properties. These activities are certainly due to the marked presence of phenolic acids, flavonoids and ascorbic acid, phytochemicals well-known to exerts these health effects *in vitro* and *in vivo*. Certainly, this is the first study, which sheds light on the main biological activities attributable to this plant complex, but further *in vitro* and *in vivo* studies are necessary to deepen the molecular mechanisms and cellular targets involved in the health activities found that could justify the use of CLJ as a safe prevention strategy for inflammatory-based diseases correlated to angiogenesis.

Another limitation of our study is certainly the low availability of *C. lumia* fruits, but as it was done previously for other fruits belonging to the *Citrus* genus, the research can shed light on new interesting species from a health point of view, paving the way also to an increase in crop production through the establishment of experimental fields.

## Data Availability Statement

The raw data supporting the conclusions of this article will be made available by the authors, without undue reservation, to any qualified researcher.

## Author Contributions

AS and DT were involved in conception and design, acquisition of data, analysis and interpretation of data. All authors performed the experiments, contribute to draft the manuscript and approved the submitted version.

## Conflict of Interest

The authors declare that the research was conducted in the absence of any commercial or financial relationships that could be construed as a potential conflict of interest.
