# Supplementary material for: Antioxidant, Anti-Inflammatory and Anti-Angiogenic Properties of Citrus lumia Juice
Source: Front Pharmacol. 2020 Dec 3;11:593506. doi: 10.3389/fphar.2020.593506 (PMC7744484; doi:10.3389/fphar.2020.593506)
Supplement: Supplementary file 1 [file presentation1.pptx]

## Slide 1
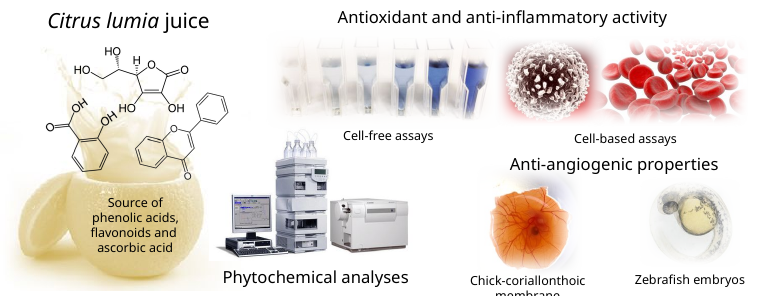

Antioxidant and anti-inflammatory activity
Citrus lumia juice
Anti-angiogenic properties
Phytochemical analyses
Zebrafish embryos
Chick-coriallonthoic membrane
Cell-free assays
Cell-based assays
Source of phenolic acids, flavonoids and
ascorbic acid
