# Supplementary material for: Antioxidant, Anti-Inflammatory and Anti-Angiogenic Properties of Citrus lumia Juice
Source: Front Pharmacol. 2020 Dec 3;11:593506. doi: 10.3389/fphar.2020.593506 (PMC7744484; doi:10.3389/fphar.2020.593506)
Supplement: Supplementary file 2 [file datasheet1.zip › Donor No. 2020-6F.pdf]

MODULO
Questionario Donatori

INFORMATIVA E CONSENSO AL TRATTAMENTO DEI DATI PERSONALI RELATIVI ALLA DONAZIONE DI SANGUE

Ai sensi del "Codice in materia di protezione dei dati personali" (Art. 13 Decreto Legislativo 196/2003), La informiamo che i Suoi dati personali, anche sensibili, saranno utilizzati esclusivamente, per finalità sanitarie volte alla valutazione dell'idoneità alla donazione di sangue ed emocomponenti e per l'adempimento degli obblighi di legge. In particolare, il servizio trasfusionale esegue sul campione di sangue i test prescritti dalla legge, inclusi i test per HIV, o altri test per la sicurezza della donazione introdotti in rapporto a specificare esigenze o a situazioni epidemiologiche, e La informerà sugli esiti degli stessi. Ove i suoi dati saranno utilizzati per studi e ricerche finalizzate alla tutela della salute, di terzi o della collettività in campo medico, biomedico ed epidemiologico, anche in relazione all'eventuale trasferimento del materiale donato e dei relativi dati ad altre strutture sanitarie, enti o istituzioni di ricerca, Le verrà fornita specifica informativa per l'acquisizione del relativo consenso del trattamento dei dati. In particolare, il materiale proveniente dalla donazione (campioni ematici/unità di emocomponenti) saranno etichettati soltanto con un codice numerico;
L'indicazione del nome, data di nascita, indirizzo, recapiti telefonici è necessaria per la Sua rintracciabilità. L'indirizzo e-mail è facoltativo e può essere utilizzato per contattarla o inviarle altre comunicazioni. Il trattamento dei dati sarà svolto in forma cartacea o elettronica, con adozione delle misure di sicurezza previste dalla legge. I suoi dati personali non saranno diffusi.
I suoi dati saranno comunicati nei casi e nei modi indicati dalla legge e dai regolamenti ai soggetti previsti, in particolare, in attuazione, della normativa sulle malattie infettive trasmissibili. Lei può in ogni momento esercitare i diritti di cui all'art. 7 del Codice (accesso, integrazione, opposizione per motivi legittimi) rivalendosi al personale indicato dal suo servizio trasfusionale.
Il mancato consenso al trattamento dei suoi dati comporterà l'esclusione dalla donazione per scopi di ricerca si sangue ed emocomponenti.

Il sottoscritto/a.[redacted].Nato/a.[redacted]
il.[redacted].residente in Via.[redacted].,n.[redacted]
Città.[redacted].CAP:[redacted]ecapiti telefonici…[redacted]
mail (facoltativo) …………………………………………………………………… acquisite le informazioni relative al trattamento dei dati personali e sensibili:

[X] **acconsento** [ ] **non acconsento**

al trattamento dei miei dati personali e sensibili

Data.[redacted].Firma del donatore.[redacted]

Ai sensi DM Salute 2 novembre 2015

Questionario donatori **Codice numerico** 2020-6F

1. Stato di salute pregresso

N.B. barrare la risposta corretta

- E' mai stato ricoverato in ospedale?.. SI ~~NO~~
Se si perché?...

- E' mai stato affetto da:

malattie autoimmuni, reumatiche osteoarticolari………………………………………... SI ~~NO~~
malattie infettive, tropicali tubercolosi……………………………………………….. SI ~~NO~~
ipertensione arteriosa, malattie cardiovascolari……………………………………… SI ~~NO~~
malattie neurologiche, svenimenti ricorrenti, convulsioni, attacchi epilettici…………… SI ~~NO~~
se si quali?...
malattie respiratorie ………………………………………………………………... SI ~~NO~~
malattie gastrointestinali, malattie del fegato, ittero………………………………….. SI ~~NO~~
malattie renali……………………………………………………………………… SI ~~NO~~

malattie del sangue o della coagulazione……………………………………………. SI ~~NO~~

malattie neoplastiche (tumori)………………………………………………………. SI ~~NO~~
diabete...………………………………………………………………………………. SI ~~NO~~

- Ha mai avuto gravidanze o interruzioni di gravidanza? …………………………… SI ~~NO~~
- Ha mai avuto shock allergico?…………………………………………………… SI ~~NO~~
- Ha mai ricevuto trasfusioni di sangue o di emocomponenti o somministrazioni di medicinali derivati dal sangue?…………………………………………………………… SI ~~NO~~
se si quali? ………………………………………………… Quando? ……………………..
- E' mai stato sottoposto a trapianto di organi, tessuti (cornea, dura madre) o di cellule?.. SI ~~NO~~
Se si quale?...
- E' vaccinato per l'epatite B? …………………………………………………… SI ~~NO~~
- I suoi genitori sono nati in paesi dell'America centrale, dell'America del Sud o in Messico?...SI ~~NO~~
Se si quale?..
- Ha trascorso un periodo superiore a 6 mesi (anche cumulativamente) nel Regno Unito nel periodo 1980-1996?...SI ~~NO~~
- E' stato trasfuso nel Regno Unito dopo il 1980?..SI ~~NO~~

2. Stato di salute attuale

- E' attualmente in buona salute? ……………………………………………………...~~SI~~ NO

- Dall'ultima donazione ha sempre goduto di buona salute?.. ~~SI~~ NO

Se no, quali malattie ha avuto e quando?..

- Ha attualmente, o ha avuto di recente, febbre o altri segni di malattia infettiva (diarrea, vomito, sindrome da raffreddamento, linfonodi ingrossati)?... SI NO
- Ha attualmente manifestazioni allergiche?... SI NO
- Si è rivolto di recente al suo medico di famiglia o ha intenzione di farlo?.............. SI NO
- Ha notato perdita di peso negli ultimi tempi?... SI NO
- Nell'ultima settimana si è sottoposto a cure odontoiatriche o ad interventi di piccola chirurgia ambulatoriale?... SI NO
- Nelle ultime 4 settimane è venuto in contatto con soggetti affetti da malattie contagiose (malattie esantematiche, mononucleosi infettiva, epatite A o altre)?................................. SI NO
- Svolge attività lavorativa che comporta rischio per la sua o per l'altrui salute o pratica hobby rischiosi?... SI NO

3. Solo per le donatrici?

- E' attualmente in gravidanza? .... SI NO
- Ha partorito negli ultimi 6 mesi? .... SI NO
- Ha avuto interruzione di gravidanza negli ultimi 6 mesi? .... SI NO

4. Farmaci, vaccini, sostanze d'abuso

- Ha assunto o sta assumendo:

farmaci per prescrizione medica .... SI NO

Se si quali?...

farmaci per propria decisione.. SI NO

sostanze, integratori/principi attivi per attività sportiva e altri prodotti acquisiti via internet o al di fuori della distribuzione autorizzata... SI NO

- Ha mai ricevuto somministrazione di ormoni della crescita o estratti ipofisari? .... SI NO
- E' stato recentemente sottoposto a vaccinazioni? .... SI NO
- Ha abusato o abusa di bevande alcoliche? .... SI NO
- Ha mai assunto o assume sostanze stupefacenti? .... SI NO

5. Esposizione al rischio di malattie trasmissibili con la trasfusione

- Ha letto e compreso le informazioni sull'AIDS, le epatiti, le altre malattie trasmissibili?.. SI NO
- Ha o ha avuto malattie sessualmente trasmissibili?.. SI NO
- Ha l'AIDS o è portatore del virus HIV o crede di esserlo?................................ SI NO
- Il suo partner è portatore del virus HIV o crede di esserlo?................................. SI NO
- Ha l'epatite B o C o è portatore del virus dell'epatite B o C o crede di esserlo? .... SI NO
- Il suo partner ha l'epatite B o C o è portatore del virus dell'epatite B o C o crede di esserlo?.. SI NO
- Dall'ultima donazione o comunque negli ultimi 4 mesi ha cambiato partner?................ SI NO
- Dall'ultima donazione e comunque negli ultimi 4 mesi ha avuto rapporti eterosessuali, omosessuali, bisessuali (rapporti genitali, orali, anali):

con partner risultato positivo ai test per l'epatite B e/o C a/o per l'AIDS?........................ SI NO
con partner che ha avuto precedenti rapporti sessuali a rischio o del quale ignora le abitudini sessuali?.. SI NO
con un partner occasionale?.. SI NO
con soggetti tossicodipendenti?.. SI NO
con scambio di denaro o droga?.. SI NO
con partner nato o proveniente da paesi esteri dove l'AIDS è una malattia diffusa e del quale non è noto se sia o meno sieropositivo?.. SI NO
- E' stato sottoposto a interventi chirurgici, indagini endoscopiche (es: colonscopia esofagogastroduodenoscopia, artroscopia, ecc.)?... SI NO

Si è sottoposto a:

a) somministrazione di sostanze per via intramuscolare o endovenosa con siringhe e/o dispositivi non sterili.. SI NO
b) agopuntura con dispositivi non monouso.. SI NO
c) tatuaggi .... SI NO
d) piercing o foratura delle orecchie.. SI NO
e) somministrazione di emocomponenti o di medicinali plasma derivati .... SI NO

Si è ferito accidentalmente con una siringa o altri strumenti contaminati dal sangue?.......... SI NO
E' stato accidentalmente esposto a contaminazione delle mucose (bocca, occhi, zone genitali) con il sangue? .... SI NO

Tali comportamenti/situazioni potrebbero averla esposta al rischio di contrarre malattie infettive trasmissibili attraverso la donazione e per tale ragione non può essere ritenuto idoneo alla donazione di sangue e/o di emocomponenti.

6. Soggiorni all'estero/provincie italiane

- E' nato, ha vissuto o vive all'estero? .... SI NO
Se si in quali paese/i?..
- Negli ultimi 6 mesi ha viaggiato all'estero, fuori dall'Europa?... SI NO
Se si in quali paese/i?..
- Ha avuto malaria o febbre inspiegata durante il viaggio in zona a rischio o entro 6 mesi dal rientro?.. SI NO
- Negli ultimi 28 giorni ha soggiornato anche solo per una notte in paesi esteri o in altre province italiane a rischio di malattia da West Nile Virus (vedi elenco allegato)?......................... SI NO
Se si, dove? ....
- Ha soggiornato negli Stati Uniti e/o Canada di recente? .... SI NO
Se si, quando?..

7. Dichiarazione

Il sottoscritto dichiara di:

- aver visonato il materiale informativo in tutte le sue parti
- aver compreso compiutamente le informazioni fornite in merito alle malattie infettive trasmissibili con particolare riguardo all'epatite B, all' epatite C e all' AIDS.
- aver risposto in maniera veritiera ai quesiti posti nel questionario essendo stato informato sul significato delle domande in esso contenute.
- essere consapevole che le informazioni fornite sul proprio stato di salute e sui propri stili di vita costituiscono un elemento fondamentale per la propria sicurezza e per la sicurezza degli operatori che utilizzeranno il campione.
- aver ottenuto una spiegazione dettagliata e comprensibile sulla procedura di prelievo proposta.
- essere stato posto in condizioni di fare domande ed eventualmente di rifiutare il consenso.
- non aver donato nell'intervallo minimo di tempo previsto per la procedura di donazione proposta.
- sottoporsi volontariamente alla donazione.
- non svolgere attività professionali o sportive di particolare rischio nelle 24 ore successive alla donazione

[X] **acconsento alla donazione** [ ] **non acconsento alla donazione**

- Autorizzare la conservazione/utilizzo di componenti o materiale biologico derivato dalla donazione per la finalità di ricerca descritte nell'informativa.

[X] **acconsento** [ ] **non acconsento**

REQUISITI PER L'ACCETAZIONE DEL DONATORE

Pressione arteriosa (mm. Hg) max ...118...... min ..65..... Peso Kg ...53...........

Sangue intero: Plasma da afaresi:

Emoglobina (g/dl)...12,5.. Emoglobina (g/dl).......................

Giudizio di idoneità: il donatore risulta

[X] Idoneo a donare il seguente emocomponente:

Tipo ...SANGUE INTERO Volume ...5 ml.............................

[..] Escluso Temporaneamente Motivo..

Durata...

[ ] Escluso Temporaneamente Motivo..
